# Clinical Effects of Jiawei Danggui Beimu Kushen Pills in the Treatment of Prostate Cancer and Their Influence on the Expression of Serum Prostate Specific Antigen

**DOI:** 10.1155/2021/1036068

**Published:** 2021-11-22

**Authors:** Haiyang Zhao, Zhiqiang Ren, Guangwen Wang

**Affiliations:** Department of Urology Surgery, The People's Hospital of Guangrao, Dongying 257300, Shandong, China

## Abstract

**Objective:**

To observe the clinical effects of Jiawei Danggui Beimu Kushen pills in treating prostate cancer and their influence on the expression of serum prostate specific antigen.

**Methods:**

A total of 234 prostate cancer patients were selected and randomly divided into observation group and control group, with 117 cases in each group. The control group was given oral bicalutamide tablets, while the observation group was treated with Jiawei Danggui Beimu Kushen pills on the basis of the control group. The treatment efficacy, IPSS score, TCM syndrome score, VAS score, quality-of-life score, and immune function of the two groups were compared before and after treatment. The serum PSA and f-PSA levels of patients before treatment and after 30 days, 90 days, and 180 days of treatment in the two groups were compared. The five-year cumulative survival rate and the incidence of adverse reactions were compared between the two groups.

**Results:**

After treatment, the total effective rate of the observation group was 88.03% (103/117), which was higher than that of the control group 69.23% (81/117); the difference was statistically significant (*P* < 0.05). After treatment, the IPSS score, TCM syndrome score, and VAS score of the two groups were reduced, and those in the observation group were lower than those in the control group; the difference was statistically significant (*P* < 0.05). After treatment, the quality-of-life scores of the two groups increased, and the observation group was higher than the control group; the difference was statistically significant (*P* < 0.05). Before treatment, there was no significant difference in serum PSA levels and f-PSA levels when comparing between the two groups of patients (*P* > 0.05). With the increase of treatment time, the two index levels of the two groups were gradually decreased. After 180 days of treatment, the two index levels of the two groups of patients were significantly lower than those before treatment, and the two index levels of the observation group were significantly lower than those of the control group; the difference was statistically significant (*P* < 0.05). After treatment, the levels of IgM and IgA in the two groups were decreased, and the level of IgG was increased. The difference between the two groups in the levels of each index before and after treatment was statistically significant (*P* < 0.05), and the difference between the two groups in the levels of each index after treatment was also statistically significant (*P* < 0.05). The five-year cumulative survival rate of the observation group was 69.23%, and the five-year cumulative survival rate of the control group was 46.15% (*P* < 0.05). There was no statistically significant difference between the two groups in the incidence of dizziness, fatigue, and gastrointestinal reactions (*P* > 0.05), but the difference in the incidence of dysuria as well as dysuria and hematuria was statistically significant (*P* < 0.05).

**Conclusion:**

Jiawei Danggui Beimu Kushen pills are effective in treating prostate cancer, which can effectively reduce the patients' IPSS score and TCM syndrome scores, relieve the pain, and improve the quality of life of patients. They also have a potential role in regulating serum PSA levels, clearing tumor lesions, reducing postoperative complications, and improving related symptoms.

## 1. Introduction

Prostate cancer (PCa) is an epithelial malignant tumor that occurs in the prostate. The clinical manifestations of prostate cancer are symptoms such as slow urinary flow, dysuria, increased nocturia, incomplete urination, urinary incontinence, and hematuria and dysuria [1]. With the intensification of the aging society, the changes in people's living habits, dietary structure, and the improvement of the level of diagnosis and treatment, the incidence of prostate cancer has been on the rise in recent years, and it has the characteristics of high mortality rate, poor prognosis, and gradual younger age. Prostate cancer has become the main cause threatening the life and health of Chinese older adults [2]. PCa mostly has a relatively insidious onset, relatively slow growth, and no obvious clinical symptoms in the early stage and is at the late clinical stage when diagnosed. With the progress of PCa, symptoms of metastasis and compression may appear, bringing about certain difficulties to the treatment of PCa [3]. Presently, prostate cancer is mainly treated with antiandrogen therapy, and Bicalutamide is currently the commonly used drug for the clinical treatment of PCa. Although Bicalutamide can have a better curative effect, it will make patients become hormone-dependent, cannot cure prostate cancer, and is prone to adverse reactions [4]. Traditional Chinese medicine practice includes rich practical experience and medical technology of ancient medical scientists. TCM treatment of prostate cancer has made great progress. In TCM, prostate cancer belongs to the categories of TCM “dysuria,” “Lin syndrome,” and “blood urine.” Treatment of prostate cancer should be through attacking toxins, promoting blood circulation and removing blood stasis, strengthening the body, and consolidating the foundation [5]. Danggui Beimu Kushen pill is one of the more promising prescriptions, which has good anti-inflammatory effects and can reduce patients' dependence on hormone therapy and improve the therapeutic efficacy of prostate cancer [6]. This study analyzed the clinical efficacy of Jiawei Danggui Beimu Kushen pills in adjuvant treatment of prostate cancer and detected their effect on the expression of serum prostate specific antigen in patients.

## 2. Materials and Methods

### 2.1. General Information

#### 2.1.1. Inclusion Criteria

The relevant diagnostic criteria of PCa in the “Guidelines for Diagnosis and Treatment of Urology” should be met [7]. The patient was pathologically confirmed by a puncture biopsy of the rectum and prostate. This study was approved by the ethics committee of the the People's Hospital of Guangrao, Dongying, Shandong, China, and the patients signed informed consent.

#### 2.1.2. Exclusion Criteria

Exclusion criteria were as follows: patients with organ dysfunction, mental illness, and immunosuppression; patients with major diseases such as hematopoietic, endocrine system or heart, liver, and kidney dysfunction; patients who are allergic to the drugs used and their ingredients; patients with other malignant tumors; patients with incomplete clinical data or lack of accuracy; and patients with a poor compliance or severe adverse reactions during treatment.

#### 2.1.3. Case Selection and Grouping

234 PCa patients at the People's Hospital of Guangrao, Dongying, Shandong, China, from January 2008 to January 2015 were selected and divided into control group (117 cases) and observation group (117 cases) according to different treatment plans. Patients in the control group were 49–76 years old, with an average age of 62.37 ± 5.48 years. The patient's course of illness was 1–7 years, with an average of 4.23 ± 1.54 years. The body mass index of patients was 22.00–26.00 kg/m^2^, with an average of 23.86 ± 0.64 kg/m^2^. There were 103 cases in T3 stage and 14 cases in T4 stage. The patients in the observation group were 50–78 years old, with an average age of 62.32 ± 5.18 years. The course of illness of patients was 1–8 years, with an average of 4.86 ± 1.63 years. The body mass index of patients was 22.00–25.00 kg/m^2^, with an average of 23.57 ± 0.68 kg/m^2^. The clinical stage of the patients was 106 cases in T3 stage and 11 cases in T4 stage. There was no statistically significant difference between the two groups of patients in general information (*P* > 0.05).

### 2.2. Treatment Methods

The control group was given Bicalutamide tablets (AstraZeneca Pharmaceutical Co., Ltd., registration number H20130043, specification: 150 mg/tablet), 150 mg/time, once daily. The observation group was given Jiawei Danggui Beimu Kushen pills on the basis of the control group. Jiawei Danggui Beimu Kushen pills are composed of Chinese medicines such as *Angelica* 10 g, *Sophora flavescens* 10 g, *Fritillaria thunbergii* 20 g, *Polygonum cuspidatum* 20 g, *Patrinia vulgaris* 10 g, Combined Spicebush Root 15 g, and Panax pseudo-ginseng powder 3 g. The medicine was boiled and concentrated, and one dose was given daily. Patients in both groups were treated for 6 months.

### 2.3. Observation Indicators and Evaluation Criteria for Curative Effects

1. The treatment effects of the two groups were compared, and the criteria for determining the efficacy were formulated according to the “Guidelines for Diagnosis and Treatment of Urology” [7]. Ineffectiveness means that there is no significant change or further aggravation of the relevant clinical symptoms after treatment, the PSA level is slightly lower than that before treatment, and the increase is <25% before treatment, or the PSA level is higher than that before treatment, and the IPSS score is lower than that before treatment by <30%. Efficient means that the clinical symptoms have improved, the PSA level has decreased by 50%, and the IPSS score has decreased by 30% to 60%. Significantly efficient means that the clinical symptoms are significantly improved, the PSA level returns to the normal range, and the IPSS score drops >60%. The total effective rate is (markedly effective + effective)/total number of cases × 100%.
2. The serum prostate specific antigen (PSA) levels and free prostate specific antigen (f-PSA) levels before treatment and after 30 days, 90 days, and 180 days of treatment in the two groups were compared. 5 mL of fasting venous blood was drawn from the patient in the morning and centrifuged for 5 minutes, and then the supernatant was removed and refrigerated for testing. Chemiluminescence immunoassay was used to determine the serum PSA and f-PSA levels of patients.
3. The IPSS score, TCM syndrome score, and VAS score were compared before and after treatment between the two groups. The clinical symptoms were assessed using the International Prostate Symptom Score (IPSS) [8], with a total score of 35 points. The higher the score, the more severe the clinical symptoms. For TCM syndrome score, refer to “Guidelines for Clinical Evaluation of Prostate Cancer in Traditional Chinese Medicine” [9]. The scoring content includes dysuria, frequent urination, incessant dripping, constipation, soreness of waist and knees, multiple pains, and five upset heat. According to the degree of severity, it is recorded as 0–3 points, and the sum of the scores of each content is the TCM syndrome points. According to the visual analogue scale (VAS) [10], the pain is evaluated, and the total score is 10 points. The higher the score, the more severe the pain.
4. The quality of life of the two groups before and after treatment is compared. The EORTC QOL-C30 self-rating scale [11] is used for evaluation, including five items: social function, cognitive function, emotional function, physical function, and role function. The total score is 100 points; the higher the score, the better the patient's quality of life.
5. The immune functions of the two groups of patients are compared. An automatic biochemical analyzer is used to detect immune function indicators. The detection approach is an immunoturbidimetric method, and the detection indicators are IgM, IgA, and IgG.
6. The five-year cumulative survival rates of the two groups are compared. There is a regular call for follow-up, the patient's survival is recorded, and the cumulative survival rate is calculated.
7. The incidences of adverse reactions between the two groups are compared. Adverse reactions include dizziness, fatigue, gastrointestinal reactions, dysuria, and hematuria and dysuria.

### 2.4. Statistical Processing

SPSS 22.0 statistical software was used for analysis. The *t*-test is performed to analyze the measurement data, which is expressed as mean ± standard deviation (SD). The *χ*^2^ test is performed to analyze the count data, which is expressed as a rate (%). *P* < 0.05 is considered statistically significant. All experiments in this article were repeated three times.

## 3. Results

### 3.1. Comparison of Clinical Efficacy between the Two Groups of Patients after Treatment

The clinical efficacy of the observation group after treatment was 88.03%, which was significantly higher than that of the control group (69.23%), and the difference was statistically significant (*X*^2^ = 12.428, *P*=0.002) (Table 1).

### 3.2. Comparison of Serum PSA Levels and f-PSA Levels before Treatment and after 30 Days, 90 Days, and 180 Days of Treatment between the Two Groups

**Figure 1:**
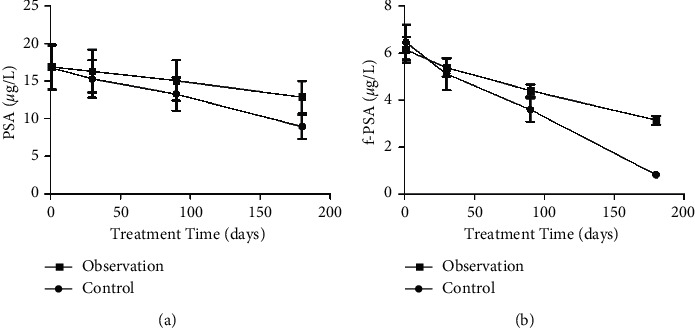
Comparison of serum PSA levels and f-PSA levels before treatment and after 30 days, 90 days, and 180 days of treatment between the two groups. (a) Comparison of serum PSA levels before treatment and after 30 days, 90 days, and 180 days of treatment between the two groups. (b) Comparison of serum f-PSA levels before treatment and after 30 days, 90 days, and 180 days of treatment between the two groups.

Before treatment, there was no significant difference in serum PSA level and f-PSA level between the two groups of patients (*P* > 0.05). With the increase of treatment time, the two index levels of the two groups of patients gradually decreased. After 180 days of treatment, the two index levels of the two groups of patients were significantly lower than those before treatment, and the index levels of the observation group were significantly lower than those of the control group; the difference was statistically significant (*P* < 0.05) (Figure 1).

### 3.3. Comparison of IPSS Scores, TCM Syndrome Scores, and VAS Scores between the Two Groups of Patients before and after Treatment

Before treatment, there was no significant difference in IPSS score, TCM syndrome score, and VAS score between the two groups of patients (*P* > 0.05). After treatment, the three scores of the two groups of patients decreased. The difference between the groups before and after treatment was statistically significant (^*∗*^*P* < 0.05), and the difference between the groups after treatment was statistically significant (^#^*P* < 0.05), as shown in Table 2.

### 3.4. Comparison of the Quality-of-Life Scores between the Two Groups of Patients before and after Treatment

Before treatment, there was no statistically significant difference in the quality-of-life scores between the two groups of patients (*P* > 0.05). (Table 3).

### 3.5. Comparison of the Immune Function of the Two Groups of Patients before and after Treatment

**Figure 2:**
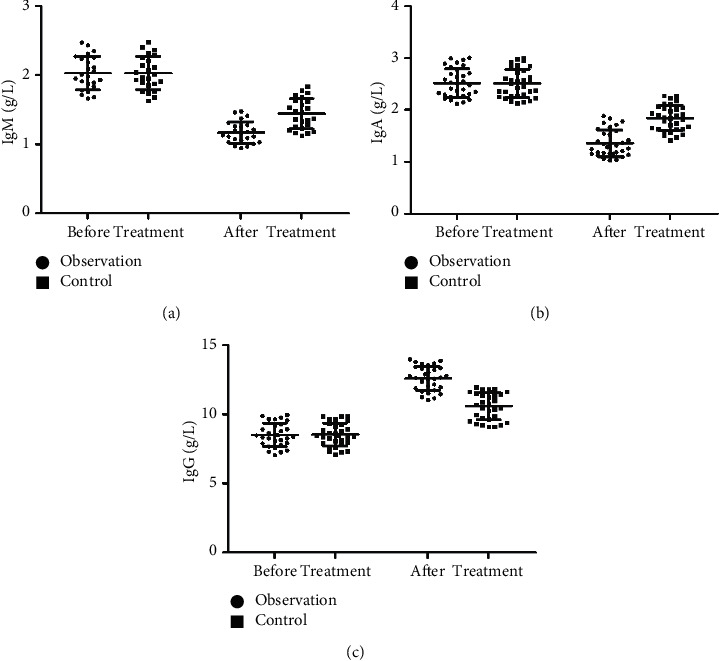
Comparison of the immune function of the two groups of patients before and after treatment. (a) Comparison of IgM levels between the two groups of patients before and after treatment. (b) Comparison of IgA levels between the two groups of patients before and after treatment. (c) Comparison of IgG levels between the two groups of patients before and after treatment.

Before treatment, there was no significant difference in IgM, IgA, and IgG levels between the two groups of patients (*P* > 0.05). After treatment, the levels of IgM and IgA in the two groups decreased, and IgG levels increased. The difference in the level of each index between the two groups before and after treatment was statistically significant (*P* < 0.05), and the difference in the level of each index between the two groups after treatment was statistically significant (*P* < 0.05) (Figure 2).

### 3.6. Comparison of the Five-Year Cumulative Survival Rates in the Two Groups of Patients

**Figure 3:**
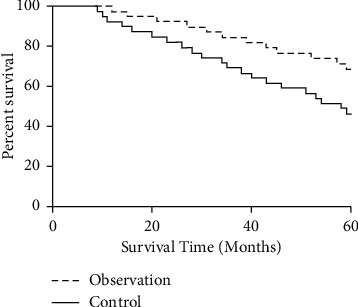
Comparison of the five-year cumulative survival rates of the two groups of patients.

The five-year cumulative survival rate of the observation group was 69.23%, and the five-year cumulative survival rate of the control group was 46.15%. The difference between the two groups was statistically significant (*P* < 0.05) (Figure 3).

### 3.7. Comparison of the Incidence of Adverse Reactions between the Two Groups of Patients after Treatment

In the observation group, there were 3 cases of dizziness, 1 case of fatigue, 4 cases of gastrointestinal reactions, 2 cases of dysuria, 2 cases of hematuria, 1 case of odynuria. In the control group, there were 5 cases of dizziness, 3 cases of fatigue, 5 cases of gastrointestinal reactions, 11 cases of dysuria, 13 cases of hematuria, and 9 cases of odynuria. There was no significant difference in the incidence of dizziness, fatigue, and gastrointestinal reactions between the two groups (*P* > 0.05). The differences in the incidence of dysuria as well as dysuria and hematuria between the two groups were statistically significant (*P* < 0.05) (Table 4).

## 4. Discussion

Prostate cancer, a urological disease, is a common and more frequent disease in modern times, which specifically refers to the presence of malignant epithelial tumors in the prostate. At present, the specific cause of PCa is not clear in clinical practice, but studies have shown that it may be related to the patient's age, region, race, genetics, environment, diet, and other factors, and it is more common in middle-aged and older adults [12, 13]. PCa has no specific symptoms in the early stage, and it is mostly in the middle and late stages when it is discovered. With the gradual enlargement of the prostate body, it can compress the urethra, causing symptoms such as dysuria and thin urine lines, and even endanger the vascular and nerve bundles and bladder, leading to impotence and hematuria, which seriously endanger the physical and mental health of patients [14]. Therefore, it is particularly necessary to find a scientific and effective treatment plan to intervene in time. The treatment of clinical PCa includes various treatment approaches such as surgery and drugs [15, 16], but surgery will cause certain wounds to patients and also affect the psychological state of patients, and some patients do not receive surgical treatment. Studies have found that [17] the growth of cancer cells in PCa patients has a certain dependence on male hormones. Bicalutamide is a class of antiandrogen drugs, which are nonsteroidal antihormonal drugs and are often used in the treatment of prostate cancer. When Bicalutamide enters the human body, it has a certain competitive effect on androgen receptor binding, reducing the production of androgens, inhibiting the proliferation and growth of tumor cells to a certain extent, and improving clinical symptoms. Bicalutamide is usually taken once a day, but it will produce androgen-dependency after taking it for a long time, which will have a certain impact on the therapeutic efficacy [18]. Regarding the various adverse reactions due to Western medicine treatment, clinicians began to seek the treatment of prostate cancer from the concepts of Chinese medicine, in order to reduce the severity and incidence of adverse reactions and improve the therapeutic effects against prostate cancer. Jiawei Danggui Beimu Kushen pills are one of the more promising prescriptions in the study of ancient Chinese medicine prescriptions for chronic prostatitis. In the prescription, *Angelica* has the effects of promoting blood circulation and removing blood stasis, moistening the intestines, and laxative. *Fritillaria thunbergii* clears away heat and reduces phlegm, relieves cough and detoxification, and has the effect of moisturizing the lungs. *Sophora flavescens* clears away heat and dampness and expels wind. Combined with *Fritillaria thunbergii*, *Sophora flavescens* can clear the lungs and dispel bladder stasis and heat. On this basis, the addition of *Polygonum cuspidatum* and *Patrinia vulgaris* is to remove the damp and heat of the lower body, and Combined Spicebush Root can relieve qi and pain, Panax pseudo-ginseng powder promotes blood circulation and removes blood stasis and relieves pain [19–22]. The combination of various medicines can make the damp heat dispel, the blood pulse cleared, and the blood stasis scattered, as well as smooth urination.

Serum PSA is the preferred specific marker for preadenocarcinoma. Serum PSA is produced in prostate epithelial cells, and its normal function is to help hydrolyze and liquefy semen clots. Serum PSA is closely related to male fertility, and the expression level is 0 in the state of no tumor. Studies have shown that PSA will be released into the blood in large quantities after PCa patients' prostate epithelium is damaged. PSA is expressed at a high level in the serum of prostate cancer patients and is positively correlated with the progression of the disease [23–25]. The results of this study showed that, before treatment, the serum PSA levels and f-PSA levels of the two groups were highly expressed. After treatment, the two index levels of the two groups decreased significantly, and in the observation group they were more significantly lower than the control group (*P* < 0.05). The results indicated that the use of Jiawei Danggui Beimu Kushen pills could significantly reduce the serum PSA level of PCa patients, inhibit tumor vitality, and improve the clinical symptoms of patients. The results of this study show that the therapeutic efficacy, IPSS score, TCM syndrome score, VAS score, and quality-of-life score in observation group are better than those of the control group. The results show that Jiawei Danggui Beimu Kushen pills have better therapeutic effects in the treatment of PCa, which can reduce the pain intensity and improve the quality of life of patients. The results of this study showed that the serum IgM and IgA levels of the observation group were lower than those of the control group, and the IgG level was higher than that of the control group, suggesting that the use of Jiawei Danggui Beimu Kushen pills to treat PCa can improve the patient's resistance and enhance the patient's immune function. The results of this study showed that the five-year cumulative survival rate of the observation group was predominantly higher than that of the control group, and the incidence of adverse reactions was lower than that of the control group, indicating that the use of Jiawei Danggui Beimu Kushen pills to treat PCa can reduce the toxic and side effects in patients using Western medicine and improve the survival time of patients, which is of great significance for the prognosis of patients.

To sum up, Jiawei Danggui Beimu Kushen pills have potential curative effects in prostate cancer treatment, significantly improving clinical symptoms, enhancing immunity, regulating PSA levels, and inhibiting tumor vitality. THey can improve the quality of life of patients, prolong the survival time of patients, and reduce the incidence of adverse reactions.

## Figures and Tables

**Table 1:** Comparison of clinical efficacy between the two groups of patients after treatment.

Groups	Number	Significantly efficient	Efficient	Ineffectiveness	Total effective rate
Observation group	117	42	61	14	103 (88.03)
Control group	117	31	50	36	81 (69.23)
*χ* ^*2*^					12.428
*P*					0.002

**Table 2:** Comparison of IPSS score, TCM syndrome score, and VAS score before and after treatment between the two groups.

Groups	Number		IPSS score	TCM syndrome score	VAS score
Observation group	117	Before treatment	19.36 ± 5.64	12.62 ± 3.46	7.56 ± 1.73
		After treatment	9.69 ± 3.37^*∗*^^#^	8.04 ± 2.11^*∗*^^#^	2.34 ± 0.96^*∗*^^#^
*t*			13.664	11.248	14.625
*P*			<0.05	<0.05	<0.05
Control group	117	Before treatment	19.78 ± 5.83	12.31 ± 3.28	7.47 ± 1.52
		After treatment	15.43 ± 4.91^*∗*^	4.69 ± 1.73^*∗*^	4.81 ± 1.15^*∗*^
*t*			7.223	6.451	5.230
*P*			<0.05	<0.05	<0.05

**Table 3:** Comparison of the quality-of-life scores of the two groups of patients before and after treatment.

Index		Observation group (117)	Control group (117)	*t*	*P*
Social function	Before treatment	52.66 ± 7.56	53.17 ± 7.84	0.982	>0.05
	After treatment	81.63 ± 6.45^*∗*^^#^	68.43 ± 6.87^*∗*^	8.231	<0.05
Affective function	Before treatment	51.41 ± 8.52	51.77 ± 8.35	0.530	>0.05
	After treatment	77.16 ± 5.43^*∗*^^#^	62.84 ± 6.72^*∗*^	7.412	<0.05
Cognitive function	Before treatment	56.53 ± 6.85	56.79 ± 6.42	0.678	>0.05
	After treatment	78.27 ± 5.46^*∗*^^#^	66.71 ± 5.97^*∗*^	10.236	<0.05
Physical function	Before treatment	53.63 ± 8.87	53.29 ± 8.66	0.374	>0.05
	After treatment	78.57 ± 6.41^*∗*^^#^	65.92 ± 6.25^*∗*^	9.623	<0.05
Role function	Before treatment	55.47 ± 6.78	55.86 ± 6.93	0.417	>0.05
	After treatment	79.41 ± 7.79^*∗*^^#^	64.36 ± 7.02^*∗*^	11.642	<0.05

**Table 4:** Comparison of the incidence of adverse reactions between the two groups of patients after treatment.

Groups	Number	Dizziness	Fatigue	Gastrointestinal reaction	Dysuria	Hematuria	Painful urination
Observation group	117	3	1	4	2	2	1
Control group	117	5	3	5	11	13	9

## Data Availability

The data used to support the findings of this study are included within the article.
